# Anti-Inflammatory Activity and Mechanism of a Lipid Extract from Hard-Shelled Mussel (*Mytilus Coruscus*) on Chronic Arthritis in Rats

**DOI:** 10.3390/md12020568

**Published:** 2014-01-27

**Authors:** Guipu Li, Yuanqing Fu, Jusheng Zheng, Duo Li

**Affiliations:** Department of Food Science and Nutrition, Zhejiang University, Hangzhou 310058, Zhejiang, China; E-Mails: guipuli@aliyun.com (G.L.); fuyuanqing@163.com (Y.F.); zhengjusheng@gmail.com (J.Z.)

**Keywords:** hard-shelled mussel lipid extract, New Zealand green-lipped mussel lipid extract, anti-inflammatory activity and mechanism, adjuvant-induced arthritis, collagen-induced arthritis

## Abstract

The present study was designed to investigate the anti-inflammatory activity and mechanism of a lipid extract from hard-shelled mussel (*Mytilus coruscus*) on adjuvant-induced (AIA) and collagen-induced arthritis (CIA) in rats. AIA and CIA rats that received hard-shelled mussel lipid extract (HMLE group) at a dose of 100 mg/kg demonstrated significantly lower paw swelling and arthritic index, but higher body weight gain than those which received olive oil (control group). Similar results were found in arthritic rats that received New Zealand green-lipped mussel lipid extract (GMLE) at the same dosage. The levels of leukotriene B_4_ (LTB_4_), prostaglandin E_2_ (PGE_2_), thromboxane B_2_ (TXB_2_) in the serum, and interleukin-1β (IL-1β), IL-6, interferon-γ (INF-γ), tumor necrosis factor-α (TNF-α) in the ankle joint synovial fluids of HMLE group rats were significantly lower than those of control group. However, the levels of IL-4 and IL-10 in HMLE group rats were significantly higher than those in the control group. Decreased mRNA expressions of matrix metalloproteinase 1 (MMP1) and MMP13, but increased tissue inhibitor of metalloproteinase 1 (TIMP1) were observed in the knee joint synovium tissues of HMLE group rats when compared with the control group. No hepatotoxicity was observed in both HMLE and GMLE group rats. The present results indicated that HMLE had a similarly strong anti-inflammatory activity as GMLE. Such a strong efficacy could result from the suppression of inflammatory mediators (LTB_4_, PGE_2_, TXB_2_), pro-inflammatory cytokines (IL-1β, IL-6, INF-γ, TNF-α) and MMPs (MMP1, MMP13), and the promotion of anti-inflammatory cytokines (IL-4, IL-10) and TIMPs (TIMP1) productions.

## 1. Introduction

Arthritis is a form of joint disorder that involves inflammation of one or more joints. It affects 10% of the world’s population. Rheumatoid arthritis (RA) and Osteoarthritis (OA) are the two most common types of arthritis. According to the data, RA affects between 0.5 and 1% of adults in the developed world with between 5 and 50 per 100,000 people newly developing the condition each year [1], while OA affects about 3.6% of the global population and nearly 27 million people in the United States [2]. The conventional therapeutic options employed in the management of RA and OA are analgesics and non-steroidal anti-inflammatory drugs (NSAIDs), but these options frequently produce suboptimal benefits and may be associated with serious gastrointestinal side effects [3]. As a result, medical researchers are looking for safer, more effective alternatives to both the traditional analgesics and NSAIDs [4].

One natural alternative for the treatment of inflammation and arthritis is the New Zealand green-lipped mussel (*Perna canaliculus*) lipid extract (GMLE). This marine mollusc lipid extract is obtained by supercritical fluid extraction (SFE) of the tartaric acid stabilized freeze-dried green-lipped mussel powder using liquefied carbon dioxide [5]. Numerous animal and clinical studies have demonstrated that GMLE has anti-inflammatory, anti-arthritic, and gastro-protective properties [6,7,8,9,10,11]. It has been proposed that the active ingredients in GLME are concentrated long chain *n*-3 polyunsaturated fatty acids (PUFAs) which can inhibit membrane arachidonic acid (AA, C20:4*n*-6) metabolism by blocking the lipoxygenase (LOX) and cyclo-oxygenase (COX) pathways, thus, decreasing prostaglandin and leukotriene synthesis and down regulating the inflammatory sequence [12,13]. *In vitro* studies have shown that GMLE can inhibit leukotriene B_4_ (LTB_4_) production in calcium ionophore-stimulated human neutrophils [6], and in interleukin-4-induced human monocytes [14]. Inhibition of prostaglandin E_2_ (PGE_2_) production in activated human macrophages has also been observed [6]. However, to our knowledge, no literature has been published on the anti-inflammatory effect of other mussel species.

China is the number one producer of mussels in the world, producing over 600,000 tons per year. Hard-shelled mussel (*Mytilus coruscus*), one main mussel species widely cultivated in coastal areas of China, is becoming the most represented mollusc in the Chinese bivalve market. Our previous research has demonstrated that hard-shelled mussel is rich in *n*-3 PUFAs, ranging from 34.2% to 36.6% of the total fatty acids [15]. Such high levels are comparable to the New Zealand green-lipped mussel, in which *n*-3 PUFAs account for 37.1% of the total fatty acids [16]. One of our pilot studies [17] has shown that the solvent extracted lipid of hard-shelled mussel has comparable anti-inflammatory properties to GMLE by decreased weight of cotton pellet in cotton pellet-induced granuloma in rats.

The aim of the present study was to investigate the anti-inflammatory activity and mechanism, and the safety of hard-shelled mussel lipid extract (HMLE), which was obtained by SFE of the tartaric acid stabilized freeze-dried hard-shelled mussel powder using liquefied carbon dioxide, on adjuvant-induced (AIA) and collagen-induced arthritis (CIA) in rats. The effects of HMLE on the paw swelling, arthritic index, and body-weight gain of AIA and CIA rats were evaluated. To evaluate the anti-inflammatory mechanism, the effects of HMLE on the levels of inflammatory mediators including LTB_4_, PGE_2_, thromboxane B_2_ (TXB_2_), and cytokines including interleukin-1β (IL-1β), IL-2, IL-4, IL-6, IL-10, tumor necrosis factor-α (TNF-α), interferon-γ (INF-γ), in the serum and ankle joint synovial fluids of AIA and CIA rats, and the mRNA expressions of matrix metalloproteinases (MMPs) including MMP1, MMP3, MMP13, and tissue inhibitor of metalloproteinase 1 (TIMP1) in the knee joint synovium tissues of CIA rats were studied. To evaluate the safety, the effects of HMLE on the hepatic enzyme activities including aspartate transaminase (AST), alanine transaminase (ALT), and alkaline phosphatase (ALP) in the serum of AIA rats were investigated. The results may provide useful information for the use of *Mytilus coruscus* as a functional food with anti-inflammatory activity or as an effective and safe alternative to tradition anti-inflammatory medication.

## 2. Results and Discussion

### 2.1. Effect of HMLE on Chronic Arthritis in AIA and CIA Rats

AIA and CIA have been widely utilized to induce arthritic immunopathological diseases that display many of the pathological features of human RA [18]. These two arthritic rat models have been used extensively to analyze the anti-inflammatory effects of newly developed drugs on chronic arthritis. Generally, chronic arthritis occurs within 14 days after immunization, and lasts for several weeks. Paw swelling, arthritis index, and body-weight gain are usually employed to evaluate the anti-inflammatory activities of drugs on AIA and CIA rats [19].

**Figure 1:**
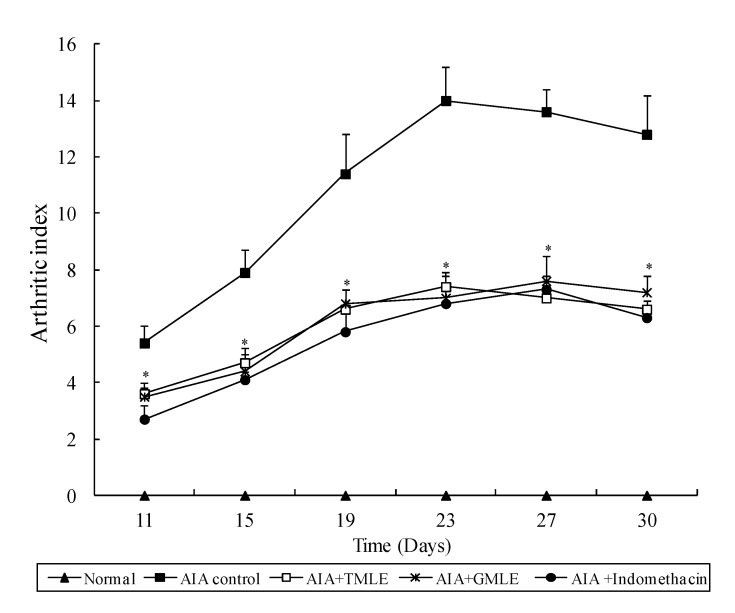
Effect of mussel extracts on the arthritic index of AIA rats. Results are presented as mean ± SD. * *p* < 0.05 compared to AIA control group. AIA, adjuvant-induced arthritis; HMLE, hard-shelled mussel lipid extract; GMLE, New Zealand green-lipped mussel lipid extract.

**Figure 2:**
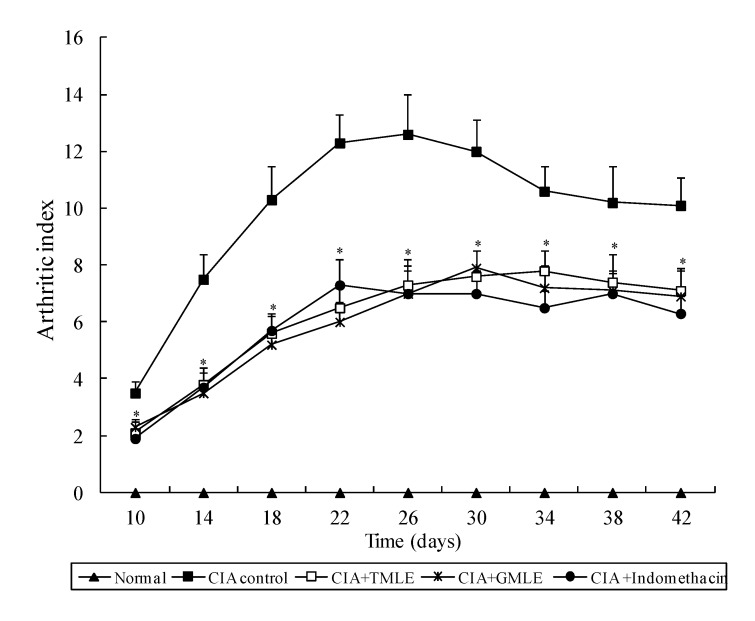
Effect of mussel extracts on the arthritic index of CIA rats. Results are presented as mean ± SD. * *p* < 0.05 compared to CIA control group. CIA, collagen-induced arthritis; HMLE, hard-shelled mussel lipid extract; GMLE, New Zealand green-lipped mussel lipid extract.

**Figure 3:**
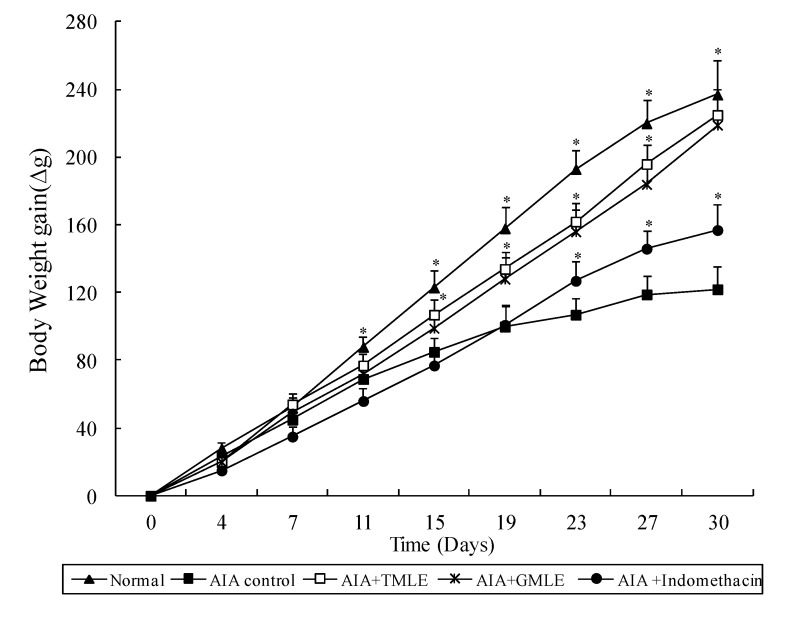
Effect of mussel extracts on the body weight gain of AIA rats. Results are presented as mean ± SD. * p < 0.05 compared to AIA control group. AIA, adjuvant-induced arthritis; HMLE, hard-shelled mussel lipid extract; GMLE, New Zealand green-lipped mussel lipid extract.

**Figure 4:**
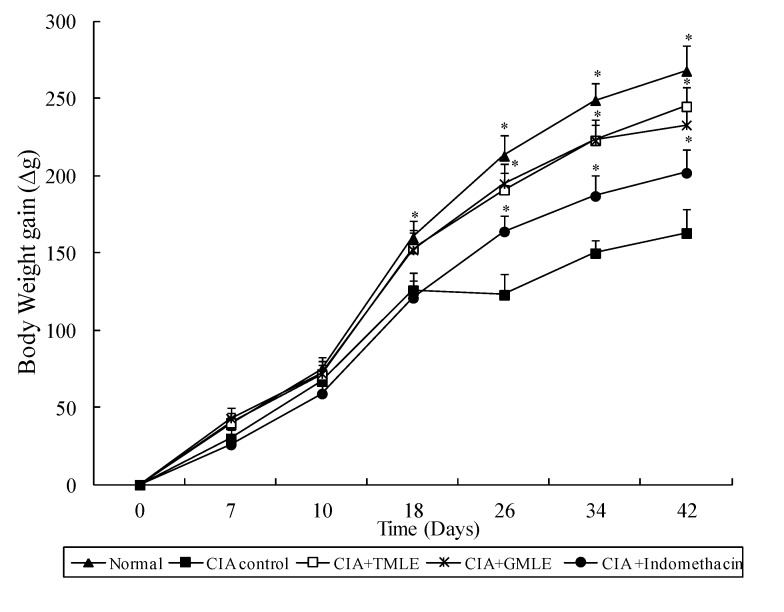
Effect of mussel extracts on the body weight gain of CIA rats. Results are presented as mean ± SD. * *p* < 0.05 compared to CIA control group. CIA, collagen-induced arthritis; HMLE, hard-shelled mussel lipid extract; GMLE, New Zealand green-lipped mussel lipid extract.

In the present study, the anti-inflammatory effects of HMLE on chronic arthritis were studied in AIA and CIA rats. Chronic arthritis, characterized by bilateral hind paw swelling was observed on day 11 and 10, and reached a peak on day 19 and 22 after the induction of AIA and CIA in rats, respectively. Therefore, the hind paw swelling and arthritis index were measured after day 11 in AIA rats and day 10 in CIA rats. As shown in Table 1 and Table 2, and Figure 1 and Figure 2, all arthritic rats that were treated with HMLE (AIA + HMLE, CIA + HMLE), GMLE (AIA + GMLE, CIA + GMLE) and Indomethacin (AIA + Indomethacin, CIA + Indomethacin) demonstrated significantly decreased hind paw swelling and lower arthritis index than the non-treated arthritic control rats (*p* < 0.05). No significant variation was found in these two parameters among the arthritic rats that received HMLE, GMLE, or Indomethacin. During arthritis development, the body-weight gain of AIA control rats after day 11 and CIA control rats after day 18 was significantly lower than that of normal rats (*p* < 0.05). HMLE + AIA (after day 15) and HMLE + CIA (after day 26) rats had significantly higher body weight gain than the AIA and CIA control rats, respectively (*p* < 0.05). Similar results were observed in the GMLE + AIA and GMLE + CIA rats (Figure 3 and Figure 4).

**Table 1:** Effect of mussel extracts on the left hind paw swelling of adjuvant-induced (AIA) rats.

Groups	Number	Dose (mg/kg)	Left Hind Paw Swelling (ΔmL)					
			Day 11	Day 15	Day 19	Day 23	Day 27	Day 30
Normal	*n* = 10	-	0.12 ± 0.01 c	0.15 ± 0.02 c	0.20 ± 0.02 c	0.25 ± 0.01 c	0.25 ± 0.00 c	0.28 ± 0.02 c
AIA control	*n* = 10	-	0.79 ± 0.20 a	1.30 ± 0.24 a	1.93 ± 0.35a	1.45 ± 0.42 a	0.98 ± 0.26 a	0.73 ± 0.17 a
AIA + HMLE	*n* = 8	100	0.43 ± 0.11 b	0.75 ± 0.20 b	0.98 ± 0.25 b	0.80 ± 0.10 b	0.56 ± 0.07 b	0.40 ± 0.14 b
AIA + GMLE	*n* = 9	100	0.46 ± 0.14 b	0.71 ± 0.17 b	1.06 ± 0.22 b	0.76 ± 0.12 b	0.53 ± 0.14 b	0.36 ± 0.08 b
AIA + Indomethacin	*n* = 7	1.5	0.39 ± 0.11 b	0.65 ± 0.13 b	0.85 ± 0.21 b	0.75 ± 0.14 b	0.48 ± 0.18 b	0.39 ± 0.10 b
*P*-value			0.000	0.000	0.000	0.000	0.000	0.000

Results are presented as mean ± SD. Values within the same column not sharing a common letter are significantly different (*p* < 0.05). AIA, adjuvant-induced arthritis; HMLE, hard-shelled mussel lipid extract; GMLE, New Zealand green-lipped mussel lipid extract.

**Table 2:** Effect of mussel extracts on the left hind paw swelling of collagen-induced arthritis (CIA) rats.

Groups	Number	Dose (mg/kg)	Left Hind Paw Swelling (ΔmL)								
			Day 10	Day 14	Day 18	Day 22	Day 26	Day 30	Day 34	Day 38	Day 42
Normal	*n* = 10	-	0.12 ± 0.00 b	0.13 ± 0.01 c	0.16 ± 0.01 c	0.22 ± 0.02 c	0.26 ± 0.01 c	0.29 ± 0.02 c	0.30 ± 0.03 d	0.30 ± 0.01 c	0.32 ± 0.02 c
CIA control	*n* = 11	-	0.25 ± 0.07 a	0.68 ± 0.13 a	0.97 ± 0.17 a	1.27 ± 0.25 a	1.20 ± 0.20 a	1.25 ± 0.27 a	1.03 ± 0.18 a	1.07 ± 0.24 a	0.96 ± 0.15 a
CIA + HMLE	*n* = 12	100	0.16 ± 0.06 ab	0.35 ± 0.07 b	0.51 ± 0.09 b	0.58 ± 0.10 b	0.65 ± 0.12 b	0.69 ± 0.10 b	0.67 ± 0.14 bc	0.64 ± 0.13 b	0.60 ± 0.11 b
CIA + GMLE	*n* = 9	100	0.14 ± 0.03 b	0.37 ± 0.06 b	0.46 ± 0.07 b	0.54 ± 0.05 b	0.62 ± 0.08 b	0.73 ± 0.14 b	0.70 ± 0.13 b	0.63 ± 0.10 b	0.56 ± 0.09 b
CIA + Indomethacin	*n* = 10	1.5	0.10 ± 0.04 b	0.36 ± 0.08 b	0.51 ± 0.11 b	0.67 ± 0.12 b	0.64 ± 0.12 b	0.60 ± 0.08 b	0.52 ± 0.09 c	0.49 ± 0.08 b	0.51 ± 0.07 b
*P*-value			0.000	0.000	0.000	0.000	0.000	0.000	0.000	0.000	0.000

Results are presented as mean ± SD. Values within the same column not sharing a common letter are significantly different (*p* < 0.05). CIA, collagen-induced arthritis; HMLE, hard-shelled mussel lipid extract; GMLE, New Zealand green-lipped mussel lipid extract.

AIA is characterized by a rapid onset and progression to polyarticular inflammation. Usually the disease is severe and leads to permanent joint malformations, including ankylosis. Symmetric joint involvement, lymphocyte infiltration, cartilage degradation, synovial hyperplasia, and T cell dependence are shared features with human RA. However, damage to cartilage is less severe than that in RA, whereas bone destruction is more prominent [20,21]. CIA is characterized by an onset of swelling of the paws of both the fore limbs and hind limbs. Swelling can persist for a few weeks, decreases gradually, and reappears, resulting in chronic arthritis with severe consequences, such as malformation of bone structures. It shares many features including symmetric joint involvement, peripheral joints affected, synovial hyperplasia, inflammatory cell infiltrate, cartilage and bone erosions, synovitis, pannus formation, and production of rheumatoid factors with human RA. However, CIA is self-limiting and not characterized by exacerbations and remission, which is the case in a large proportion of patients with RA [21,22]. No animal model of arthritis can represent all the pathology of human RA. Thus, it is better to employ more than one classic arthritic animal model to study the pre-clinical therapeutic effects of anti-arthritic agents. To our knowledge, this was the first study to investigate the anti-inflammatory activity of HMLE using both AIA and CIA animal models. HMLE (100 mg/kg) demonstrated a strong anti-inflammatory effect by diminishing the hind paw swelling and arthritis index, and improving the body weight gain in both AIA and CIA rats. Many previous animal studies have demonstrated that GMLE can reduce inflammation in arthritic rats [6,12] and dogs [7,23]. In addition, numerous clinical trials have shown a strong therapeutic activity of GMLE in patients suffering from RA and OA [11,24]. In the present study, HMLE displayed similar anti-inflammatory efficacy to GMLE in rats with AIA and CIA. 

### 2.2. Effect of HMLE on the Levels of Inflammatory Mediators in the Serum of AIA and CIA Rats

All arthritic control rats demonstrated significantly higher levels of PGE_2_, LTB_4_, and TXB_2_ in the serum compared with normal rats (*p* < 0.05). HMLE + AIA and HMLE + CIA rats had significantly lower levels of PGE_2_, LTB_4_, and TXB_2_ than the AIA and CIA control rats, respectively (*p* < 0.05). Similar results were observed in the GMLE + AIA and GMLE + CIA rats. Such results indicated that HMLE at the dose of 100 mg/kg can strongly inhibit the excessive production of inflammatory mediators, and the efficacy is similar to that of GMLE at the same dosage (Table 3 and Table 4).

**Table 3:** Effect of mussel extracts on the LTB_4_, PGE_2_, and TXB_2_ levels in the serum of AIA rats.

Groups	Number	Dose (mg/kg)	LTB_4_ (ng/L)	PGE_2_ (µg/L)	TXB_2_ (ng/L)
Normal	*n* = 10	-	10.37 ± 1.09 c	1.03 ± 0.13 c	38.64 ± 6.18 c
AIA control	*n* = 10	-	15.68 ± 1.42 a	3.76 ± 0.37 a	75.43 ± 10.75 a
AIA + HMLE	*n* = 8	100	11.86 ± 1.83 b	2.38 ± 0.27 b	41.77 ± 5.13 c
AIA + GMLE	*n* = 9	100	11.24 ± 1.25 bc	2.03 ± 0.18 b	48.42 ± 7.07 b
AIA + Indomethacin	*n* = 7	1.5	15.33 ± 1.72 a	0.74 ± 0.15 d	27.68 ± 4.10 d
*P*-value			0.000	0.000	0.000

Results are presented as mean ± SD. Values within the same column not sharing a common letter are significantly different (*p* < 0.05). AIA, adjuvant-induced arthritis; HMLE, hard-shelled mussel lipid extract; GMLE, New Zealand green-lipped mussel lipid extract; LTB_4_, leukotriene B_4_; PGE_2_, prostaglandin E_2_; TXB_2_, thromboxane B_2_.

**Table 4:** Effect of mussel extracts on the LTB_4_, PGE_2_, and TXB_2_ levels in the serum of CIA rats.

Groups	Number	Dose (mg/kg)	LTB_4_ (ng/L)	PGE_2_ (µg/L)	TXB_2_ (ng/L)
Normal	*n* = 10	-	15.41 ± 1.37 ^c^	0.84 ± 0.13 ^c^	40.33 ± 4.51 ^d^
CIA control	*n* = 11	-	28.26 ± 3.83 ^a^	3.26 ± 0.46 ^a^	104.15 ± 15.57 ^a^
CIA + HMLE	*n* = 12	100	20.75 ± 3.16 ^b^	1.64 ± 0.23 ^b^	73.26 ± 9.27 ^b^
CIA + GMLE	*n* = 9	100	18.42 ± 2.73 ^b^	1.82 ± 0.19 ^b^	58.41 ± 8.36 ^c^
CIA + Indomethacin	*n* = 10	1.5	26.24 ± 3.02 ^a^	0.64 ± 0.11 ^d^	43.16 ± 4.26 ^d^
*P*-value			0.000	0.000	0.000

Results are presented as mean ± SD. Values within the same column not sharing a common letter are significantly different (*p* < 0.05). CIA, collagen-induced arthritis; HMLE, hard-shelled mussel lipid extract; GMLE, New Zealand green-lipped mussel lipid extract; LTB_4_, leukotriene B_4_; PGE_2_, prostaglandin E_2_; TXB_2_, thromboxane B_2_.

PGE_2_, TXB_2_, and LTB_4_ are key inflammatory mediators, which are derived from AA through the COX and LOX pathways in cell membranes of mammals [25]. They are involved in modulating the intensity and duration of inflammatory responses, have cell- and stimulus-specific sources and frequently have opposing effects. PGE_2_ has a number of pro-inflammatory effects, including increasing vascular permeability, vasodilation, blood flow, and local pyrexia and potentiation of pain caused by other agents. It also promotes the production of some MMPs and stimulates bone resorption [26]. LTB_4_ increases vascular permeability, enhances local blood flow, is a potent chemotactic agent for leucocytes, induces release of lysosomal enzymes, and enhances release of reactive oxygen species and inflammatory cytokines, such as TNF-α, IL-1β and IL-6 [27]. High levels of PGE_2_, TXB_2_, and LTB_4_ are found in the serum and joint tissues in rat models of arthritis [28], and in the synovial fluids of patients with active RA [29]. Theoretically, any substance that can decrease the production of PGE_2_, TXB_2_, and LTB_4_, can be considered as a potential anti-inflammatory agent.

In the present study, both AIA and CIA rats, which received HMLE and GMLE, demonstrated decreased levels of serum PGE_2_, TXB_2_, and LTB_4_ compared with non-treated arthritic control rats. This result suggests that HMLE and GMLE exert anti-inflammatory activities partially through the suppression of PGE_2_, TXB_2_, and LTB_4_ production in the serum of arthritic rats. Previous research demonstrated that GMLE inhibits LTB_4_ biosynthesis by stimulated human polymorphonuclear leukocytes and monocytes, and PGE_2_ production by activated human macrophages *in vitro* [6,14]. This anti-inflammatory activity was largely associated with concentrated *n*-3 PUFAs in the GMLE. It has been widely reported that the anti-inflammatory effect of *n*-3 PUFAs (especially EPA and DHA) appears to reflect their role as competitive substrates for eicosanoid formation from AA [22,25]. Although both *n*-3 and *n*-6 PUFAs share the same enzymes for PGs and LTs biosynthesis, the products generated from each are molecularly and physiologically distinct. When *n*-3 PUFAs are the COX and LOX substrates, PGE_3_, TXB_3_, and LTB_5_, which are much less pro-inflammatory, are produced instead of AA-derived PGE_2_, TXB_2_, and LTB_4_ [28,29,30].

### 2.3. Effect of HMLE on the Levels of Inflammatory Cytokines in the Ankle Joint Synovial Fluids of AIA and CIA Rats

Seven inflammatory cytokines including IL-1β, IL-2, IL-4, IL-6, IL-10, IFN-γ, and TNF-α were analyzed in all experimental rats. In the case of AIA (Table 5), significantly increased levels of IL-1β, IL-2, IL-6, IFN-γ, and TNF-α in the ankle joint synovial fluids were observed in the AIA control rats compared with the normal rats (*p* < 0.05). HMLE + AIA rats displayed significant lower levels of IL-1β, IL-6, IFN-γ, and TNF-α, but higher levels of IL-4 and IL-10 in the ankle joint synovial fluids than the AIA control rats (*p* < 0.05). No significant variation was found in the levels of all analyzed inflammatory cytokines between the HMLE + AIA and GMLE + AIA rats. With regard to CIA (Table 6), significantly increased levels of IL-1β, IL-2, IL-6, IL-10, IFN-γ, and TNF-α, but decreased level of IL-4 in the ankle joint synovial fluids were observed in the CIA control rats compared with normal rats (*p* < 0.05). HMLE + CIA rats displayed significantly lower levels of IL-1β, IL-6, IFN-γ, and TNF-α, but a higher level of IL-10 in the ankle joint synovial fluids than the CIA control rats (*p* < 0.05). Similar results were observed in the GMLE + CIA rats.

TNF-α, IL-1β, IL-2, IL-6, and IFN-γ are important pro-inflammatory cytokines implicated in the pathogenesis of arthritis [31]. These cytokines play a fundamental role in the processes that cause inflammation, articular destruction, cartilage degradation, bone erosion, and the comorbidities associated with RA [32]. Increased levels of TNF-α, IL-1β, IL-2, IL-6 and IFN-γ are found in the joint tissues of arthritic rats and patients suffering from RA [31,32]. IL-4 and IL-10 are key anti-inflammatory cytokines involved in arthritic diseases. Previous studies have shown they can inhibit a range of inflammatory mediators including PGE_2_, LTB_4_, and pro-inflammatory cytokines, including TNF-α, IL-1β, IL-6, IL-8, IL-12, IFN-γ, at both protein and mRNA levels *in vitro* and *in vivo*, thus, ameliorate the progression of inflammation seen in arthritis [33,34]. It is well known that an imbalance between pro- and anti-inflammatory cytokine activities favors the induction of autoimmunity, chronic inflammation and thereby joint damage in arthritis [31].

The present study showed that arthritic rats which received HMLE and GMLE at a dose of 100 mg/kg had decreased IL-1β, IL-6, IFN-γ, and TNF-α, but increased IL-4 and IL-10 levels in the ankle joint synovial fluids compared with non-treated arthritic control rats. These results indicate that HMLE and GMLE exert anti-inflammatory activities by the blockage of IL-1β, IL-6, IFN-γ, and TNF-α production, and by the promotion of IL-4 and IL-10 production in the ankle joint synovial fluids of arthritic rats.

**Table 5:** Effect of mussel extracts on the IL-1β, IL-2, IL-4, IL-6, IL-10, IFN-γ, and TNF-α levels in the ankle joint synovial fluids of AIA rats.

Groups	Number	Dose (mg/kg)	IL-1β (ng/L)	IL-2 (ng/L)	IL-4 (ng/L)	IL-6 (ng/L)	IL-10 (ng/L)	IFN-γ (ng/L)	TNF-α (ng/L)
Normal	*n* = 10	-	29.71 ± 4.28 c	5.23 ± 1.96 c	20.36 ± 3.72 b	56.16 ± 13.58 c	11.35 ± 3.73 c	15.83 ± 4.64 b	49.71 ± 10.31 d
AIA control	*n* = 10	-	53.66 ± 10.66 a	10.18 ± 2.74 a	15.72 ± 3.55 b	154.23 ± 39.63 a	16.57 ± 5.24 c	25.66 ± 5.83 a	381.45 ± 89.45 a
AIA + HMLE	*n* = 8	100	40.37 ± 10.32 b	9.45 ± 3.31 ab	28.13 ± 6.48 a	95.46 ± 26.73 b	26.43 ± 7.36 b	18.37 ± 4.36 b	125.27 ± 28.37 b
AIA + GMLE	*n* = 9	100	38.16 ± 7.15 b	10.61 ± 3.86 a	27.29 ± 7.35 a	89.37 ± 24.75 b	24.21 ± 5.83 b	16.21 ± 5.73 b	100.35 ± 23.66 bc
AIA + Indomethacin	*n* = 7	1.5	38.42 ± 8.06 b	7.48 ± 2.43 bc	28.76 ± 6.76 a	81.36 ± 17.26 b	34.79 ± 7.21 a	17.45 ± 3.94 b	87.34 ± 15.84 c
*P*-value			0.000	0.000	0.000	0.000	0.000	0.000	0.000

Results are presented as mean ± SD. Values within the same column not sharing a common letter are significantly different (*p* < 0.05). AIA, adjuvant-induced arthritis; HMLE, hard-shelled mussel lipid extract; GMLE, New Zealand green-lipped mussel lipid extract; IL-1β, interleukin-1β; IL-2, interleukin-2; IL-4, interleukin-4; IL-6, interleukin-6; IL-10, interleukin-10; INF-γ, interferon-γ; TNF-α, tumor necrosis factor-α.

**Table 6:** Effect of mussel extracts on the IL-1β, IL-2, IL-4, IL-6, IL-10, IFN-γ, and TNF-α levels in the ankle joint synovial fluids of CIA rats.

Groups	Number	Dose (mg/kg)	IL-1β (ng/L)	IL-2 (ng/L)	IL-4 (ng/L)	IL-6 (ng/L)	IL-10 (ng/L)	IFN-γ (ng/L)	TNF-α (ng/L)
Normal	*n* = 10	-	37.83 ± 9.43 d	8.45 ± 2.68 b	33.24 ± 5.25 a	79.26 ± 15.46 c	47.93 ± 10.71 c	15.35 ± 3.68 b	43.61 ± 7.89 c
CIA control	*n* = 11	-	90.61 ± 20.17 a	17.46 ± 5.34 a	20.67 ± 4.76 c	168.55 ± 37.38 a	78.35 ± 18.73 b	27.61 ± 6.04 a	162.29 ± 33.75 a
CIA + HMLE	*n* = 12	100	61.33 ± 15.35 b	18.54 ± 4.79 a	22.82 ± 5.33 c	111.37 ± 25.45 b	126.68 ± 30.26 a	18.41 ± 5.43 b	83.77 ± 18.38 b
CIA + GMLE	*n* = 9	100	57.75 ± 13.21 bc	15.13 ± 4.72 a	21.57 ± 5.69 c	119.64 ± 28.72 b	132.47 ± 28.66 a	18.23 ± 4.93 b	97.64 ± 20.56 b
CIA + Indomethacin	*n* = 10	1.5	47.28 ± 8.24 cd	10.85 ± 3.53 b	26.83 ± 4.97 b	111.84 ± 20.25 b	84.38 ± 18.54 b	16.76 ± 3.16 b	80.63 ± 20.72 b
*P*-value			0.000	0.000	0.000	0.000	0.000	0.000	0.000

Results are presented as mean ± SD. Values within the same column not sharing a common letter are significantly different (*p* < 0.05). CIA, collagen-induced arthritis; HMLE, hard-shelled mussel lipid extract; GMLE, New Zealand green-lipped mussel lipid extract; IL-1β, interleukin-1β; IL-2, interleukin-2; IL-4, interleukin-4; IL-6, interleukin-6; IL-10, interleukin-10; INF-γ, interferon-γ; TNF-α, tumor necrosis factor-α.

### 2.4. Effect of HMLE on the mRNA Expression Levels of Matrix Metalloproteinase in the Knee Joint Synovium Tissues of CIA Rats

**Figure 5:**
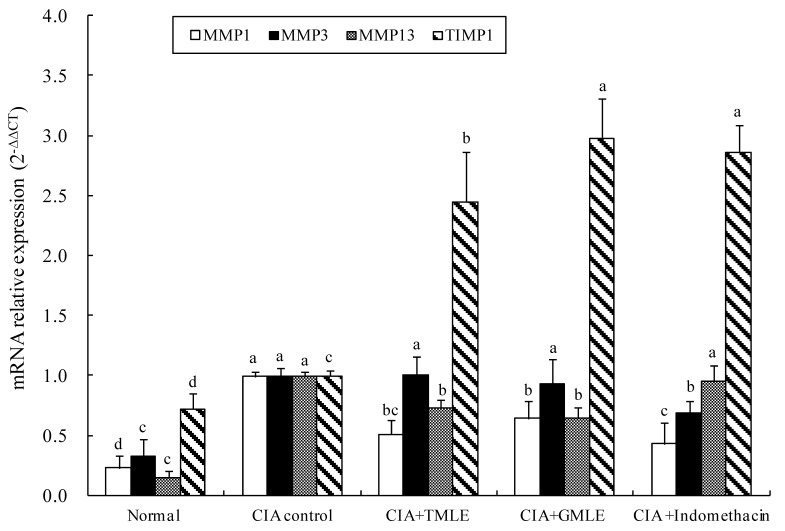
Effect of mussel extracts on the MMP1, MMP3, MMP13, and TIMP1 mRNA expression levels in the knee joint synovium tissues of CIA rats. Results are presented as mean ± SD. Values in the bars of same type not sharing a common letter are significantly different (*p* < 0.05). CIA, collagen-induced arthritis; HMLE, hard-shelled mussel lipid extract; GMLE, New Zealand green-lipped mussel lipid extract; MMP1, matrix metalloproteinase 1; MMP3,matrix metalloproteinase 3; MMP13,matrix metalloproteinase 13; TIMP1, tissue inhibitor of metalloproteinase 1.

The mRNA expressions of MMP1, MMP3, MMP13 and TIMP1 in the knee joint synovium tissues of CIA rats were analyzed (Figure 5). Compared to the normal group, significantly increased MMP1, MMP3, MMP13 and TIMP1 mRNA expression levels were observed in the CIA control group (*p* < 0.05). Both the HMLE + CIA and GMLE + CIA groups demonstrated significantly lower MMP1 and MMP13, but higher TIMP1 mRNA expression levels than the CIA control group (*p* < 0.05). No significant difference in the mRNA expressions of MMP1, MMP3, MMP13, and TIMP1 was observed between the HMLE + CIA and GMLE + CIA groups.

MMPs are a large group of enzymes responsible for matrix degradation. They contribute to joint destruction in RA and OA by directly degrading the cartilage, tendon, bone and indirectly promoting angiogenesis (formation of new blood vessels) [35]. Among MMPs, the collagenases, MMP1 and MMP13 have predominant roles in RA and OA because they have a rate-limiting role in the process of collagen degradation. MMP1 is produced primarily by the synovial cells that line the joints, and MMP13 is a product of the chondrocytes that reside in the cartilage [36]. Overexpression of MMPs such as MMP1, MMP2, MMP3, MMP9, and MMP13, has been found in the joint tissues of arthritic rats and patients suffering from RA and OA [36,37]. It is reported that inhibition of MMPs gene expression can provide new opportunities for the development of therapeutics to prevent the joint destruction seen in arthritis [36]. TIMPs are specific inhibitors that bind MMPs in a 1:1 stoichiometry. Four TIMPs, including TIMP1, TIMP2, TIMP3, and TIMP4, have been identified in vertebrates, and their expression is regulated during development and tissue remodeling [38]. Under pathological conditions associated with unbalanced MMP activities, changes of TIMP levels are considered to be important because they directly affect the level of MMP activity [39].

The present study demonstrated that HMLE, at a dose of 100 mg/kg, could up-regulate the MMP1 and MMP13, but down-regulate the TIMP1 mRNA expressions in the knee joint synovium tissues of CIA rats. The efficacy was similar to GMLE at the same dose. Such findings indicate that HMLE and GMLE exert anti-inflammatory activities partially by the inhibition of MMP1 and MMP13, and the stimulation of TIMP1 mRNA expression in the knee joint synovium tissues of arthritic rats.

### 2.5. Effect of HMLE on the Hepatic Enzyme Activities in the Serum of AIA Rats

Table 7 shows the AST, ALT, and ALP activities in the serum of normal and AIA treated rats. Compared to the normal group, significantly higher ALT and ALP activities (*p* < 0.05), but similar AST activities were observed in the AIA control group. Both, AIA + HMLE and AIA + GMLE groups, demonstrated significantly lower ALT and ALP activities than the AIA control group (*p* < 0.05). No significant variation was found in AST, ALT, and ALP activities among AIA + HMLE, AIA + GMLE and normal groups. AIA + Indomethacin group displayed significantly higher ALT and ALP activities than all other groups.

**Table 7:** Effect of mussel extracts on the aspartate transaminase (AST), alanine transaminase (ALT), and alkaline phosphatase (ALP) activities in the serum of AIA rats.

Groups	Number	Dose (mg/kg)	AST (U/L)	ALT (U/L)	ALP (U/L)
Normal	*n* = 10	-	48.74 ± 5.29 ab	133.4 ± 21.2 c	208.6 ± 31.7 c
AIA control	*n* = 10	-	45.68 ± 8.35 ab	276.5 ± 35.3 b	275.3 ± 36.5 b
AIA + HMLE	*n* = 8	100	43.86 ± 7.38 b	161.4 ± 27.2 c	217.7 ± 25.3 c
AIA + GMLE	*n* = 9	100	47.81 ± 7.97 ab	148.2 ± 21.8 c	196.4 ± 30.1 c
AIA + Indomethacin	*n* = 7	1.5	58.53 ± 10.12 a	374.8 ± 51.6 a	412.4 ± 60.3 a
*P*-value			0.017	0.000	0.000

Results are presented as mean ± SD. Values within the same column not sharing a common letter are significantly different (*p* < 0.05). AIA, adjuvant-induced arthritis; HMLE, hard-shelled mussel lipid extract; GMLE, New Zealand green-lipped mussel lipid extract; AST, aspartate transaminase; ALT, alanine transaminase; ALP, alkaline phosphatase.

AST, ALT, and ALP are three most important hepatic enzymes involved in the hepatotoxicity. Increased serum activities of these enzymes are extensively used as indicators of anti-inflammatory, therapy-induced hepatic lesion in rheumatic arthritis [40,41]. In the present study, elevated ALT and ALP activities were found in the serum of AIA control group when compared with the normal group. This result indicates that hepatic injuries occur in rats during the development of AIA. Several studies have shown a correlation between the development of an inflammatory response and the activation of hepatic enzymes in AIA [42,43]. Both, HMLE and GMLE treatments at the dose (100 mg/kg) used in our study lead a large decrease in ALT and ALP activities in the serum of AIA rats, and the activities were revert to the same rates seen in normal rats. This observation suggests that HMLE and GMLE have no hepatotoxicity. Inversely, they can protect rats from hepatic injuries, which are caused by the arthritic disease. Indomethacin treatments at a dose of 1.5 mg/kg largely increased the serum ALT and ALP activities in AIA rats, indicating that Indomethacin have strong hepatotoxicity.

## 3. Experimental Section

### 3.1. Hard-Shelled Mussel Collection and HMLE Preparation

Hard-shelled mussels of commercial size were collected in November, 2011, from Shengsi Islands in Zhejiang province. After collection, all mussels were immediately transported under refrigeration (4 °C) to the Department of Food Science and Nutrition, Zhejiang University, China. Mussel shells were removed and the fleshy portion were minced and blended with 3% of tartaric acid, vacuum freeze-dried and homogenized to powder, and then stored at −20 °C until lipid extraction.

HMLE was extracted by the procedure of CO_2_-SFE from the tartaric acid stabilized freeze-dried hard-shelled mussel powder, according to the method of Macrides and Kalafatis with minor modification [5]. Briefly, mussel powder (600 g) was charged to the Super Critical Fluid Extraction Unit (Nantong Hua-an Super Critical Extraction Co., Ltd., Jiangsu, China). Supercritical-CO_2_ was delivered at a flow rate of 25 kg/h for two hours. The extract temperature was set at 50 °C and the extract pressure at 35 MPa. The evaporator parameters were set at 40 °C and 7 MPa. The lipid extract presented as amber oil, and was stored under nitrogen at −80 °C in amber vials to minimize autoxidation. Fatty acid composition of HMLE was measured by gas chromatography (GC) at different time occasions (0, 30, 60, 90 days) during the storage (Table 8). The results showed no significant variation in the contents of most fatty acids, indicating that HMLE was stable.

**Table 8:** Fatty acid composition (% of total fatty acids) of hard-shelled mussel lipid extract (HMLE) at different time occasions during the storage.

Fatty Acid	Storage Time				*P*-value
	0 Days	30 Days	60 Days	90 Days	
C12:0	0.26 ± 0.13 b	0.65 ± 0.09 a	0.63 ± 0.17 a	0.38 ± 0.11 b	0.014
C14:0	2.31 ± 0.19	2.43 ± 0.15	2.46 ± 0.22	2.37 ± 0.20	0.783
C15:0	0.68 ± 0.09	0.75 ± 0.06	0.79 ± 0.13	0.64 ± 0.14	0.391
C16:0	20.05 ± 1.35	20.90 ± 1.17	21.54 ± 1.23	22.03 ± 1.50	0.350
C17:0	2.24 ± 0.12 b	2.53 ± 0.30 ab	2.28 ± 0.11 b	2.69 ± 0.22 a	0.049
C18:0	6.31 ± 0.44	7.43 ± 0.11	6.79 ± 0.41	7.11 ± 0.67	0.168
C23:0	1.41 ± 0.07 a	1.32 ± 0.27 ab	0.96 ± 0.15 b	1.56 ± 0.29 a	0.046
Total SFA	33.26 ± 2.08	36.01 ± 1.92	35.45 ± 2.20	36.78 ± 2.56	0.308
C15:1	0.34 ± 0.14 ab	0.41 ± 0.05 a	0.49 ± 0.13 a	0.17 ± 0.09 b	0.030
C16:1*n*-7	7.28 ± 0.42	7.17 ± 0.37	7.46 ± 0.32	7.24 ± 0.34	0.810
C17:1	4.14 ± 0.37	3.62 ± 0.31	3.82 ± 0.38	3.93 ± 0.34	0.388
C18:1*n*-9	1.65 ± 0.45	1.31 ± 0.08	1.39 ± 0.15	1.26 ± 0.13	0.298
C18:1*n*-7	2.88 ± 0.18	2.77 ± 0.16	2.87 ± 0.14	2.75 ± 0.12	0.641
C20:1	5.81 ± 0.35	6.20 ± 0.43	6.17 ± 0.50	5.87 ± 0.47	0.620
Total MUFA	22.10 ± 1.58	21.48 ± 1.28	22.20 ± 1.07	21.22 ± 1.69	0.802
C18:3*n-*3	1.48 ± 0.15	1.78 ± 0.21	1.63 ± 0.12	1.67 ± 0.09	0.182
C18:4*n-*3	3.52 ± 0.26	3.84 ± 0.27	3.32 ± 0.32	3.57 ± 0.21	0.205
C20:5*n-*3	13.06 ± 1.02	12.50 ± 0.57	12.79 ± 0.62	11.75 ± 0.87	0.280
C22:5*n-*3	0.43 ± 0.14 b	0.63 ± 0.08 b	1.14 ± 0.18 a	1.33 ± 0.25 a	0.001
C22:6*n-*3	18.15 ± 1.10	16.70 ± 0.92	16.34 ± 1.12	16.18 ± 1.52	0.239
Total *n-*3 PUFA	36.64 ± 2.51	35.45 ± 1.46	35.22 ± 1.93	34.50 ± 1.85	0.628
C18:2*n-*6	2.21 ± 0.27	2.24 ± 0.20	1.91 ± 0.17	1.84 ± 0.37	0.225
C20:2*n-*6	0.52 ± 0.14 a	0.18 ± 0.05 b	0.18 ± 0.04 b	0.47 ± 0.23 a	0.027
C20:3*n-*6	0.45 ± 0.26 ab	0.14 ± 0.07 b	0.23 ± 0.09 b	0.56 ± 0.14 a	0.040
C20:4*n-*6	2.83 ± 0.31	2.60 ± 0.23	2.55 ± 0.29	2.37 ± 0.21	0.274
C22:3*n-*6	1.42 ± 0.58	1.17 ± 0.09	1.24 ± 0.12	1.48 ± 0.34	0.667
C22:4*n-*6	0.18 ± 0.11 b	0.37 ± 0.12 ab	0.58 ± 0.19 a	0.61 ± 0.22 a	0.043
C22:5*n-*6	0.40 ± 0.04	0.35 ± 0.07	0.38 ± 0.17	0.22 ± 0.14	0.304
Total *n-*6 PUFA	8.01 ± 0.74	7.05 ± 0.49	7.07 ± 0.54	7.55 ± 0.58	0.232
Total PUFA	44.65 ± 2.72	42.50 ± 1.86	42.29 ± 1.91	42.05 ± 2.12	0.479

Results are presented as mean ± SD (*n* = 3). Values within the same row not sharing a common letter are significantly different (*p* < 0.05). SFA, saturated fatty acid; MUFA, monounsaturated fatty acid; PUFA, polyunsaturated fatty acid.

### 3.2. Other Materials and Chemicals

GMLE was supported by Pharmalink Marketing Australia Pty. Ltd. Olive oil (Olivoilá Co., Ltd., Shanghai, China) was purchased from a local supermarket in Hangzhou. Indomethacin (Jiangxi Pharmaceutical Co., Ltd., Jiangxi, China) was purchased from a local drugstore in Hangzhou.

### 3.3. Experimental Animals

Male Sprague-Dawley rats (130–140 g) were obtained from the Animal Resources Centre (Zhejiang University, Hangzhou, China). They were maintained in standard laboratory cages, each had five rats, in moderate humidity (50% ± 5%), at constant temperature (22 ± 1 °C) in a 12-h light–dark cycle room. All animals had free access to food and water during the experimental period. The experiment protocol was approved by the Ethics Committee, College of Biosystem Engineering and Food Science, Zhejiang University.

### 3.4. Induction, Treatment and Measurement of AIA in Rats

Seventy male Sprague-Dawley rats were randomly divided into six groups, including normal group (*n* = 10), AIA group (*n* = 15), AIA + HMLE group (*n* = 15), AIA + GMLE group (*n* = 15), and AIA + Indomethacin group (*n* = 15). On day 0, AIA was induced with 0.1 mL Feund’s complete adjuvant (10 mg/mL heat inactivated *Mycobacterium tuberculosis* suspension in paraffin oil) by intradermal injection into the right hind paws of all groups except normal group, which were injected with 0.1 mL paraffin oil only [44]. From day 1 to 30 after immunization, both normal and AIA groups of rats received olive oil by gavage at a volume of 1 mL/kg daily, while AIA + HMLE, AIA + GMLE and AIA + Indomethacin groups of rats received the same volume of olive oil containing HMLE, GMLE, and Indomethacin, respectively. The concentration of HMLE, GMLE, and Indomethacin in the olive oil used for administration was 100, 100, and 1.5 mg/mL, respectively. Thus, the dose of HMLE, GMLE and Indomethacin was 100, 100, and 1.5 mg/kg, respectively. In order to evaluate the therapeutic effects of HMLE on AIA rats, three different pre-clinical parameters, including hind paw swelling, arthritis index, and body-weight gain, were measured as apparent indicators of arthritic symptoms by the research workers who were blind to the rat groups. The hind paw swelling (∆mL) was expressed as an increase in left hind paw volume by subtracting the paw volume at day 0 from that at days 11, 15, 19, 23, 27, and 30, respectively. The arthritis index was measured at days 11, 15, 19, 23, 27, and 30, and was scored as the sum of tail and four paws, graded on a scale of 0–4 each (total score = 20): for tail—0, no inflammatory nodule; 1, 1 inflammatory nodule; 2, 2–3 inflammatory nodules; 3, 3–5 inflammatory nodules; 4, more than 5 inflammatory nodules; for paw—0, no swelling; 1, involvement of isolated phalanx joint; 2, involvement of phalanx joint and digits; 3, involvement of the entire region down to the ankle; 4, involvement of entire paw, including ankle [45]. The body-weight gain (∆g) was measured by subtracting the body weight of rats at day 0 from that at days 4, 7, 11, 15, 19, 23, 27, and 30 respectively. The rats (except normal ones), of which the arthritis index was below 4 during the whole period of experiment, were considered as not suffering from chronic arthritis. The data of these rats were discarded.

### 3.5. Induction, Treatment, and Measurement of CIA in Rats

Apart from the induction of arthritis and measurement time of arthritic symptoms, all other procedures of the CIA experiment, including the subgroups of rats, drug administrations and arthritic parameter measurement methods, were the same as those of the AIA experiment. On day 0, all arthritic rats were intradermally injected with 0.5 mL Feund’s complete adjuvant emulsion containing 1 mg/mL bovine type II collagen (Sigma-Aldrich Corporation, St. Louis, MO, USA) at the back (four sites) and base of the tail (one site), followed by a booster injection seven days later using the same method to induce CIA. However, the normal rats were injected with 0.5 mL sterilized physiological saline only. During the experiment, the hind paw swelling and arthritic index of all rats were measured at days 10, 14, 18, 22, 26, 30, 34, 38, and 42, and the body weight gain was monitored at days 0, 7, 10, 18, 26, 34, and 42 after the first injection.

### 3.6. Sample Collection

At the end of the experiments (day 30 and 42 after immunization for AIA rats and CIA rats, respectively), rats were fasted overnight and sacrificed. Blood samples were collected into labeled vacuum tubes (5 mL, Gongdong Medical Technology Co., Ltd., Zhejiang, China) and processed into serum. Both hind paws of each rat were transected on proximal tibia and a 2-mm incision was made lateral to the ankle joint region of each paw after removal of skin and hair. Synovial fluids of the ankle joints were collected by lavaging the viscous fluid under the ankle and around the joints, followed by flushing two times, each time flushing with 50 µL of ice-cold physiological saline [46]. After collection, serum and ankle joint synovial fluids were immediately stored at −80 °C in closed, labeled plastic tubes until inflammatory mediators, cytokines and hepatic enzyme activities were analyzed. Knee joint synovium tissues were collected by microdissection of both knee joints of rats and immediately frozen in liquid nitrogen until MMPs and TIMPs mRNA expression quantifications [46].

### 3.7. Measurement of Inflammatory Mediators and Cytokines in Serum and Ankle Joint Synovial Fluids of Rats

Levels of inflammatory mediators (PGE_2_, LTB_4_, TXB_2_) and cytokines (IL-1β, IL-2, IL-4, IL-6, IL-10, IFN-γ, TNF-α) from serum and ankle joint synovial fluids were determined by the research workers who were blind to the rat groups using ELISA Kits (Nanjing Jiancheng Technology Co., Ltd., Nanjing, China), according to the manufacturer’s instructions. Samples were diluted using phosphate buffered saline (pH 7.4) prior to ELISA analysis if necessary.

### 3.8. Measurement of MMPs and TIMPs mRNA Expressions in the Knee Joint Synovium Tissues of Rats

Total RNA in the knee joint synovium tissues of rats were extracted by using the RNAiso Plus Total RNA Extraction Kits (Takara Biotechnology (Dalian) Co., Ltd., Liaoning, China), and the cDNA synthesis reaction were performed using 2 µg extracted pure RNA and MMLV Reverse Transcriptase 1st-Strand cDNA Synthesis Kits (Epicentre^®^ Biotechnologies Company, Madison, WI, USA) according to the manufacturer’s instructions.

The mRNA expressions of MMP1, MMP3, MMP13, and TIMP1 were determined by quantitative real-time reverse transcription-polymerase chain reaction (RT-PCR) using the LightCycler 1536 Real-time PCR Detection System (Hoffmann-La Roche Ltd., Swiss, Switzerland). The GAPDH (housekeeping gene) was used as the internal control gene to normalize the variations in cDNA content. The primer pairs for the target genes (MMP1, MMP3, MMP13, and TIMP1) and internal control gene (GAPDH) were designed and synthesized by Takara Biotechnology (Dalian) Co., Ltd., as shown in Table 9. The PCR reaction was carried out in a 20 µL volume of PCR medium containing 10 µL of SYBR Premix Ex Taq™ II (Takara Biotechnology (Dalian) Co., Ltd., Liaoning, China), 2 µL of synthesised cDNA, 1.6 µL of gene-specific primer (10 µM), and 6.4 µL of dH_2_O. The PCR precycling steps were 1 min at 95 °C (pre-denaturation), followed by 5 s at 95 °C (denaturation) and 25 s at 60 °C (annealing and elongation) for 40 cycles. At the end of the thermal cycles a dissociation protocol was performed, starting at 60 °C and measuring fluorescence with 0.5 °C increments, to ensure that a single product was detected for each primer pair. An automatic threshold over background was selected, and the cycle corresponding to its surpass (C_T_) was defined. For each reaction, C_T_s for different target genes were normalized with the corresponding GAPDH C_T_ by substraction (ΔC_T_). The relative quantification of gene expressions was performed using the 2^−ΔΔC^_T_ method as previously described [47]. The results were expressed as the mRNA expressions of target genes in the normal or treated arthritic rats, compared with those in non-treated arthritic control rats. The whole measurements were done by the research workers who were blind to the treated rat groups.

**Table 9:** Primer sequences used for RT-PCR analysis.

Gene Name	Primer Sequence (5'–3')	Size
MMP1	Forward: GAGAAAGAAGACAAAGGCAA Reverse: AGCCACATCAGGCACTCC	164 bp
MMP3	Forward: CGGTGGCTTCAGTACCTT Reverse: CCTCCTCCCAGACCTTCA	140 bp
MMP13	Forward: GCCAGAACTTCCCAACCA Reverse: ACCCTCCATAATGTCATACCC	115 bp
TIMP1	Forward: CTCTGGCATCCTCTTGTTG Reverse: CGCTGGTATAAGGTGGTCT	156 bp
GAPDH	Forward: GCAAGTTCAACGGCACAG Reverse: GCCAGTAGACTCCACGACAT	140 bp

### 3.9. Measurement of Hepatic Enzyme Activities in the Serum of Rats

The activities of hepatic enzymes including AST, ALT, and ALP in the serum were determined by the research workers who were blind to the rat groups using AST, ALT, and ALP activity assay kits (Beijing Kemeidongya Biotechnology Co., Ltd., Beijing, China), according to the manufacturer’s instructions. Samples were diluted using phosphate buffered saline (pH 7.4) prior to activity analysis if necessary.

### 3.10. Statistical Analysis

All data were reported as means and standard deviations. Comparisons of the pre-clinical arthritic parameters and levels of inflammatory factors in serum and joint tissues of different groups of rats were done by one-way analysis of variance (ANOVA). The Fisher’s Least Significant Difference (LSD) procedure was used to test for differences between means at a 5% significance level. Before statistical analyses, data were checked for normal distribution and variance homogeneity. Whenever these assumptions were violated, before statistical analyses, data were log 10 transformed to ensure normality and homogeneity. In most cases, after the transformation, both assumptions were satisfied. All analyses were conducted using SPSS 16.0 (SPSS Inc., Chicago, IL, USA).

## 4. Conclusions

The present study showed that HMLE at a dose of 100 mg/kg possessed a similarly strong anti-inflammatory activity compared with GMLE, by diminishing the hind paw swelling and arthritis index, and by improving the body weight gain in both AIA and CIA rats. This strong efficacy may be associated with the down-regulation of inflammatory mediators (LTB_4_, PGE_2_, TXB_2_), pro-inflammatory cytokines (IL-1β, IL-6, IFN-γ, TNF-α) productions and MMPs (MMP1, MMP13) mRNA expressions, and the up-regulation of anti-inflammatory cytokines (IL-4, IL-10) productions and TIMP1 mRNA expressions in the serum and joint tissues of arthritic rats. No hepatotoxicity was observed in AIA rats that received HMLE and GMLE. Further research is warranted to investigate the clinical therapeutic effect and mechanism of HMLE on chronic inflammation of patients suffering from RA and OA.
